# Imagerie de l'encéphalomyélite aiguë disséminée: étude de 22 cas

**DOI:** 10.11604/pamj.2019.34.41.15312

**Published:** 2019-09-19

**Authors:** Mohamed Abdellaoui, Souad Chaouir, Hassan Boumdin

**Affiliations:** 1 Mohamed Abdellaoui, Service d'Imagerie Médicale Hôpital Militaire Mohammed V, Rabat, Maroc

**Keywords:** ADEM, IRM, diagnostic, évolution, ADEM, MRI, diagnosis, outcome

## Abstract

L'Encéphalomyélite aiguë disséminée (ADEM) est une cause rare d'encéphalite de l'adulte, caractérisée par des lésions inflammatoires de la substance blanche du système nerveux central (SNC). Le tableau clinique initial peut mimer un tableau sévère d'infection du SNC avec fièvre, encéphalopathie, crises convulsives, ou un tableau de sclérose en plaque. Le but de notre travail est de rapporter les caractéristiques épidémiologiques, cliniques, radiologiques, thérapeutiques et évolutives de l'ADEM, aussi évaluer l'apport des séquences IRM dans le diagnostic, le suivi et le pronostic de la maladie. Il s'agit d'une étude rétrospective des dossiers cliniques et radiologiques d'ADEM sur 11 ans (janvier 2006-janvier 2017) portant sur 22 cas suivis au service de neurologie et explorés au niveau du service d'imagerie médicale de l'Hôpital Militaire d'Instruction Mohammed V de Rabat. L'âge moyen des patients était de 35 ans [12-57ans]. Une notion d'infection récente était retrouvée dans 31%, une vaccination récente dans 4%. La symptomatologie était dominée par un déficit neurologique focal avec un pourcentage de 72%. La TDM était normale dans 78%. L'IRM a montré un hyper signal FLAIR de la SB sus et sous tentoriel dans 70%, sans restriction de la diffusion dans la totalité des cas, avec rehaussement des lésions dans 27%, l'atteinte de la moelle cervicale estimait à 68%. L'évolution clinique et radiologique était favorable dans la totalité des cas.

## Abstract (en)

Acute disseminated encephalomyelitis (ADEM) is a rare cause of encephalitis in adults, characterized by inflammatory lesions in the white substance of the central nervous system (CNS). Initial clinical presentation may mimic severe CNS infection with fever, encephalopathy, seizures, or multiple sclerosis. The purpose of our study was to report the epidemiological, clinical, radiological, therapeutic and evolutionary features of ADEM and to determine the role of MRI sequences in the diagnosis, monitoring and prognosis of this disease. We conducted a retrospective study of the clinical and radiological records of 22 patients with ADEM followed up in the Department of Neurology over a period of 11 years (January 2006- January 2017). These patients underwent medical imaging at the Mohammed V Military Teaching Hospital, de Rabat. The average age of patients was 35 years (12-57 years). A recent infection was found in 31% of patients, while 4% of patients had recently undergone vaccination. Symptoms were dominated by focal neurologic deficit (72%). CT scan was normal in 78%. MRI showed hyperintense subtentorial and supratentorial white substance on FLAIR sequences in 70% of patients, without diffusion restriction in all cases, with enhancement of the lesions in 27% of cases and involvement of the cervical spinal cord in 68% of cases. Clinical and radiological outcome was favorable in all cases.

## Introduction

L'encéphalomyélite aiguë disséminée ou «acute disseminated encephalomyelitis» (ADEM) est une maladie inflammatoire démyélinisante multifocale du système nerveux central (SNC). Son mécanisme est auto-immun suite à une vaccination ou à un épisode infectieux le plus souvent viral [1]. Cette pathologie est plus fréquente chez l'enfant que chez l'adulte [2]. Elle est de présentation clinique variable, caractérisée par un tableau d'encéphalopathie avec des signes neurologiques multifocaux nécessitant en général une admission en réanimation. Il existe souvent un intervalle libre entre le facteur déclenchant éventuel et l'apparition des signes cliniques, dont la durée varie de 2 à 30 jours. L'imagerie cérébrale, essentiellement l'imagerie par résonance magnétique (IRM), joue un rôle majeur dans le diagnostic positif de cette maladie. Elle montre des lésions diffuses ou multifocales de la substance blanche du SNC. L'EMAD a un pronostic plutôt bon si la prise en charge est adéquate, il pose un problème de diagnostic différentiel avec la sclérose en plaques (SEP). La distinction entre les 2 entités est souvent difficile et, parfois, seule l'épreuve du temps la permet. A travers une étude rétrospective de cas d'ADEM, explorés dans nos services, les objectifs de notre travail sont de rapporter les caractéristiques épidémiologiques, cliniques, radiologiques, thérapeutiques et évolutives de l'ADEM, et d'évaluer l'apport des nouvelles séquences IRM dans le diagnostic, le suivi et le pronostic des encéphalomyélites aiguës disséminées (ADEM).

## Méthodes

Notre étude porte sur les cas d'encéphalomyélite aiguë disséminée, suivis au service de neurologie et explorés au niveau du service d'imagerie médicale de l'Hôpital Militaire d'Instruction Mohammed V. Il s'agit d'une étude rétrospective des dossiers cliniques et radiologiques d'ADEM sur une période de 11 ans (2006 à 2017). Les critères d'inclusion étaient tous les patients chez qui le diagnostic d'ADEM a été confirmé sur les données cliniques, biologiques et en IRM. Les critères d'exclusion étaient les cas dont le diagnostic d'ADEM n'a pas été confirmé et/ou les patients qui n'ont pas d'IRM dans leurs dossiers. Nous avons retenu 22 cas dans cette étude.

## Résultats

**Caractéristiques cliniques et biologiques** (Tableau 1): la population étudiée était composée de 8 hommes et 14 femmes. L'âge moyen était de 35 ans avec des extrêmes de 12 ans et 57 ans.

**Tableau 1:** Caractéristiques cliniques et biologiques de l’encéphalomyélite aiguë disséminée (n=22)

	Nombre (n)	Pourcentage (%)
Infection récente	n=7	31%
Vaccination récente	n=1	4%
**Motif d’hospitalisation**		
Fièvre	n=3	13%
Céphalées	n=6	27%
Convulsions	n=1	4%
Troubles du comportement	n=0	0%
**Signes physiques**		
Troubles de la conscience	n=2	9%
Syndrome méningé	n=1	4%
Déficit moteur	n=16	72%
Trouble sensitif	n=13	59%
Troubles de la marche	n=2	9%
Atteinte des paires crâniennes	n=6	27%
Troubles sphinctériens	n=11	50%
**Ponction lombaire**		
Formule normale	n=16	72%
Méningite lymphocytaire	n=6	27%
Suivi clinique moyen	**8 mois** [extrêmes de 2 mois_2 ans]	

**Caractéristiques en imagerie:** tous les patients avaient une IRM encéphalique et de contrôle (critère d'inclusion). Les examens d'imagerie réalisés chez les patients sont résumés sur le (Tableau 2).

**Tableau 2:** Examens d’imagerie pratiqués chez les patients atteints d’encéphalomyélite aigu ë disséminée (n=22)

Imagerie cérébrale initiale	Effectifs	pourcentage
TDM	n=4	18%
IRM encéphalique	n=22	100%
IRM médullaire complémentaire	n=16	72%
**Imagerie de contrôle**	n=22	100%
TDM	n=0	0%
IRM	n=22	100%

**Figure 1:**
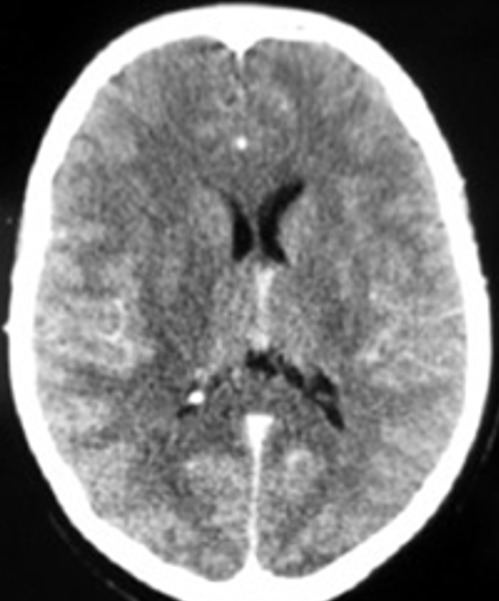
TDM cérébrale coupe axiale: montrant des hypodensités de la substance blanche pariéto occipitale prédominant du côté droit, non rehaussées après injection du produit de contraste, avec discret effet de masse sur le ventricule latéral homolatéral

**Figure 2:**
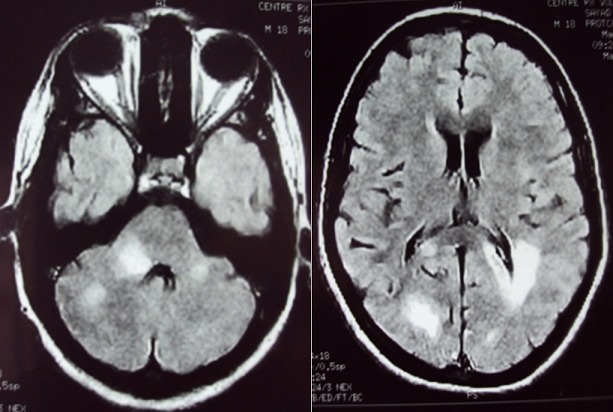
IRM cérébrale, coupes axiales, en séquences pondérées Flair: montrant des lésions d'allure démyélinisantes multifocales et de grande taille, touchant la substance blanche sus et sous-tentorielle

**Figure 3:**
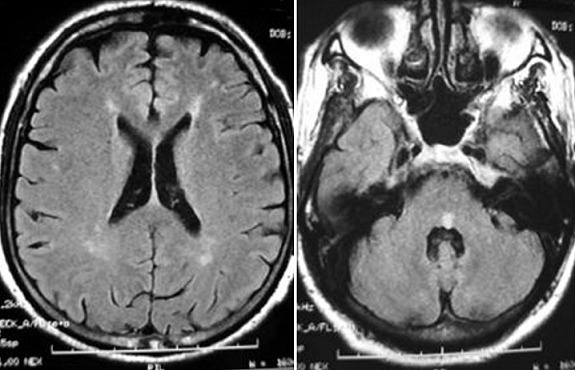
IRM cérébrale, coupes axiales, séquence Flair: montrant plusieurs lésions punctiformes touchant la substance blanche périventriculaire et la région protubérantielle (7 mm de grand axe), sans œdème périlésionnel

**Figure 4:**
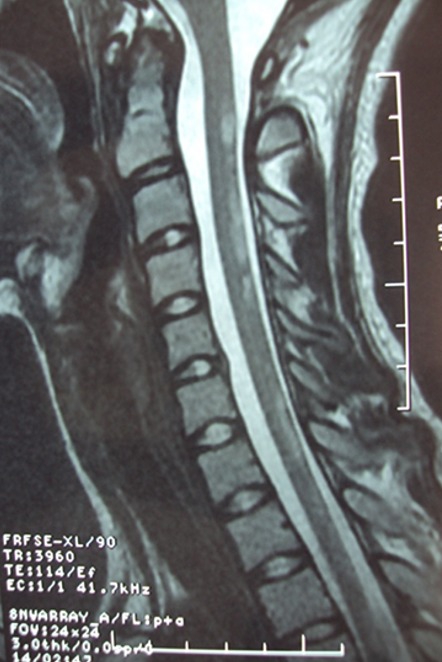
IRM médullaire, coupe sagittale, séquence T2: montrant un hypersignal médullaire étendu cervico dorsal

**Résultats en imagerie** (Tableau 3): dans notre série la TDM cérébrale était normale dans la majorité des cas et montrait des lésions hypodenses multifocales de la substance blanche dans quatre cas (Figure 1). L'IRM montrait des lésions multifocales en hyper T2 FLAIR de la substance blanche sus et sous tentoriel (Figure 2, Figure 3) sans restriction de la diffusion et sans rehaussement, avec atteinte de la moelle cervicale dans la 68% des cas (Figure 4)

**Tableau 3:** Encéphalomyélite aiguë disséminée: lésions observées en imagerie (n=22)

Lésions encéphaliques	Nombre (n)	Pourcentage (%)
TDM cérébrale		
Lésions hypodenses **(** **figure 1****)**	n=4	18%
**IRM Cérébrale : *lésion en***		
Isosignal T1	n=4	18%
Hyposignal T1	n=18	81%
Hypersignal T2, FLAIR **(** **figure 2****)**	n=22	100%
Diffusion avec un ADC bas	n=0	0%
**Siège des anomalies**		
Sus tentoriel **(** **figure 3****)**	n=2	9%
Sous tentoriel **(** **figure 4****)**	n=5	22%
Sus et sous tentoriel	n=15	68%
**Atteinte de la substance**		
Substance blanche **(figure 5)**	n=18	81%
Substance grise	n=2	9%
Cortex	n=2	9%
Noyaux gris centraux	n=1	4%
Substance blanche et grise	n=4	18%
Atteinte médullaire associée **(figure 6)**	n=15	68%
**Rehaussement après injection de PC**	n=6	27%
Annulaire	n=1	4%
En motte	n=3	13%
Nodulaire	n=2	9%

**Aspects évolutifs et pronostiques** (Tableau 4)**:** le délai moyen des contrôles IRM était de 62 jours, dans notre série l'évolution clinique est favorable la totalité des cas, avec disparition des lésions à l'IRM de contrôle dans la majorité des cas.

**Tableau 4:** Aspects évolutifs et pronostiques de l’encéphalomyélite aiguë disséminée (n=22)

Aspects évolutifs et pronostic	Nombre (n)	Pourcentage (%)
**Résultats de l’imagerie de contrôle**		
Disparition des lésions	n=18	81%
Régression	n=4	18%
Aggravation	n=0	0%
Séquelles	n=0	0%
**Evolution clinique**		
Favorable	n=19	86%
Séquelles modérées	n=3	13%
Séquelles importantes	n=0	0%
Décès	n=0	0%
**Pronostic**		
Bon: évolution favorable ou séquelles modérées	n=22	100%
Mauvais: séquelles importantes ou décès	n=0	0%

## Discussion

L'ADEM une maladie inflammatoire démyélinisante du système nerveux central. Elle est habituellement secondaire à une infection virale, bactérienne ou à une vaccination, mais peut aussi apparaître spontanément. Elle consiste en une inflammation auto-immune aboutissant à la destruction des gaines de myéline dans la substance blanche, et présente à cet égard des similitudes (physiopathologiques, cliniques et paracliniques) avec les poussées de sclérose en plaques. L'ADEM est plus fréquente chez l'enfant que chez l'adulte [2]. Une prépondérance masculine est rapportée dans plusieurs cohortes pédiatriques avec un rapport sexe féminin/masculin entre 0,6 et 0,8 [3-5]. Cette tendance est moins nette chez l'adulte (la sex-ratio observée est de 1,3 à 1,7) [2]. Il semble exister une prédominance saisonnière, avec un pic en hiver et au printemps [4, 6]. Il existe souvent un intervalle libre entre le facteur déclenchant éventuel (vaccination, infection) et l'apparition des signes cliniques, dont la durée varie de deux à 30 jours [7]. Les troubles neurologiques apparaissent dans les jours suivant la résolution de l'infection. Le début est brutal ou rapidement progressif, les symptômes se développent en quelques heures à quelques jours, en moyenne 4,5 jours. Un tableau encéphalitique associant troubles de la conscience, convulsions, fièvre et raideur méningée est fréquent. Les troubles de conscience sont retrouvés dans 19 à 69% des cas [3-8]. Les cas les plus sévères peuvent se compliquer de coma avec signes de décérébration (dans notre série 9% des cas ont présenté un trouble de conscience). La raideur méningée est signalée dans 5 à 44% des cas suivant les séries [6, 5] (5% dans notre série). La fièvre est plus fréquemment retrouvée chez l'enfant (43-52%) que chez l'adulte (15%) [3, 2, 9] (13% dans notre série). Les convulsions sont rapportées dans 4 à 30% des cas chez l'adulte [3, 4] (5% dans notre série) et dans 13 à 35% des cas chez l'enfant [7, 9]. Des signes focaux déficitaires sont fréquents. L'hémiplégie est notée dans environ 75% des cas (72% dans notre série). On retrouve un syndrome pyramidal uni- ou bilatéral dans 60 à 95% des cas, une atteinte des paires crâniennes dans 23 à 50% des cas [3, 6] (27% dans notre série). L'atteinte visuelle, particulièrement évocatrice, est caractérisée par une neuropathie optique uni- ou bilatérale et est retrouvée chez 7 à 28% des patients [5, 8]. L'atteinte médullaire est rapportée de manière variable (2 à 43% des cas), de nombreux travaux citent une fréquence d'environ 20 à 25% [3, 4, 7, 10]. Elle se manifeste par une para- ou tétraplégie aiguë avec abolition des réflexes, troubles sensitifs et vésicosphinctériens et évolue secondairement vers une spasticité. Cette atteinte était présente dans 50% des cas de notre série. L'analyse du LCR est fondamentale et permet tout d'abord d'exclure une méningoencéphalite infectieuse nécessitant un traitement spécifique. Le LCR peut montrer des anomalies non spécifiques à type de pléïocytose lymphocytaire associée à une hyperprotéinorachie. La synthèse intrathécale d'immunoglobulines (Ig G) est retrouvée en proportion variable, elle est en général transitoire [11]: 38% des cas chez l'adulte [2], 3 à 29% des cas chez l'enfant [3, 9]. Le LCR est strictement normal dans 19 à 33% des cas chez l'adulte [2, 8]. Dans notre série, l'analyse du LCR était normale dans 72% des cas, et montrait une méningite lymphocytaire dans 28% des cas. L'imagerie montre une atteinte multifocale, souvent bilatérale mais asymétrique; peut toucher le cerveau, le cervelet, le tronc cérébral et la moelle épinière, l'atteinte prédomine à la SB sous corticale, aux centres semi-ovales et à la jonction SB-SG; la SB péri-ventriculaire peut être touchée également (30-60%), et des noyaux gris centraux (dans notre série 80% atteinte de la SB, et 18% substance blanche et grise). Le scanner initial est normal dans environ 60% des cas, ou montrer des de lésions hypodenses multifocales de la SB, et peuvent apparaitre dans les 5 à 14 jours après le début des symptômes neurologiques. Le rehaussement est possible sous forme punctiforme, nodulaire ou annulaire. Dans notre série la TDM cérébrale était normale dans 82% des cas et montrait des lésions hypodenses multifocales de la substance blanche dans 18% des cas. L'IRM cérébrale avec injection de gadolinium est l'examen de choix. L'ADEM est caractérisée par des lésions apparaissant en hypersignal sur les séquences pondérées en T2 et fluid-attenuated inversion recovery (FLAIR). Les lésions sont typiquement multiples, de grande taille (>1 à 2 cm), disséminées, mal délimitées, asymétriques. Elles prédominent dans la substance blanche au niveau des régions sous-corticales, des centres semi-ovales et à la jonction substance grise corticale-substance blanche des hémisphères cérébraux. Les lésions périventriculaires sont rapportées avec une fréquence de 30 à 60%, les lésions du corps calleux sont moins fréquentes [4, 9, 12]. Une atteinte diffuse de la substance blanche était notée dans 30% des cas. Les lésions sous-tentorielles sont rapportées dans environ 35% des cas [2]. Les lésions multifocales de la substance blanche étaient retrouvées chez 81% des patients de notre série, 9% sus tentoriel, 22% sous tentoriel et 66% sus et sous tentoriel. L'atteinte de la substance grise profonde (thalamus, noyaux gris centraux) est notée dans 15 à 60% des cas [2, 6] (23% des patients de notre série). Les lésions sont rehaussées par l'injection de gadolinium dans 6 à 40% des (27% des patients de notre série), la prise de contraste dépendant du stade de l'inflammation [7, 8, 10]. La prise de contraste méningée est plus rare. L'IRM initiale peut être normale, devant être répétée quelques jours après le début des symptômes [13].

L'atteinte médullaire est retrouvée dans 9 à 28% des cas et observée essentiellement au niveau thoracique [3, 10]. Dans notre série 68% des patients présentaient une atteinte médullaire sur l'IRM. La moelle est élargie, œdémateuse, la prise de contraste est variable. L'évolution est marquée par une régression partielle des lésions dans 25 à 53% des cas (19% des patients de notre série) et une résolution complète des lésions dans 35 à 75% des cas [3, 14, 15] (81% des patients de notre série). La spectroscopique (SpectroMR) montre des anomalies peu spécifiques, au sein des hypersignaux T2, on observe des taux bas de N-acétyl-aspartate (NAA) reflétant une destruction neuronale et des taux élevés de lactates, témoignant d'un métabolisme anaérobie. La plupart des auteurs décrivent au stade aigu un hypersignal en séquence de diffusion avec un ADC bas [4, 14]. L'ADC s'élève au stade subaigu de la maladie [16]. Peu d'études se sont intéressées à la valeur pronostique de l'ADC. Certains auteurs ont démontré l'absence de corrélation entre l'ADC calculé et le pronostic de l'ADEM [16]. Notre série n'a pas montré d'hypersignal diffusion pour la totalité des patients. Le diagnostic différentiel se fait essentiellement avec la sclérose en plaque, l'IRM montre des lésions bien limitées « en plaques », siégeant en périaqueducale, du corps calleux (contrairement à l'ADEM) et de la SB péri-ventriculaire, l'Atteinte de la SG est rare (possible en cas d'ADEM). Le pronostic à long terme de la SEP est péjoratif comparativement à celui de l'ADEM. L'approche thérapeutique repose sur les traitements immunomodulateurs. Les traitements les plus utilisés sont les corticoïdes (CT) intraveineux, les immunoglobulines polyvalentes (IgIV) et les échanges plasmatiques (EP). L'ADEM présente une bonne évolution clinique dans 70 à 90% des cas [8]. Dans notre série l'évolution clinique est favorable la totalité des cas. L'évolution des lésions radiologiques se fait le plus souvent vers la régression et la disparition dans les 6 premiers mois [8, 12, 13]. Cette régression radiologique est parfois retardée par rapport à l'amélioration clinique [10]. La persistance des lésions IRM ou l'apparition de nouvelles lésions au-delà des 6 premiers mois suivant l'épisode initial est corrélé à un mauvais pronostic [12]. Dans notre série, le contrôle par IRM des lésions d'ADEM a démontré une disparition de ces lésions dans 18 cas, une régression dans 4 cas Il n'y a pas de consensus bien établi concernant le nombre et le délai des contrôles IRM. Cependant, dans la littérature, il est conseillé de pratiquer au moins 2 examens IRM après la première IRM de contrôle normale et ceci sur une période de surveillance de 5 ans à partir de l'épisode initial [13]. Dans notre étude, le délai moyen de contrôle des ADEM a été de 62 jours.

## Conclusion

L'encéphalomyélite aiguë disséminée, est une maladie inflammatoire démyélinisante du système nerveux central (SNC), elle est plus fréquente chez l'enfant que chez l'adulte. Elle est habituellement secondaire à une infection virale, bactérienne ou à une vaccination, les signes focaux déficitaires et l'atteinte médullaire prédominent le tableau clinique, la ponction lombaire peut montrer des anomalies non spécifiques à type de méningite lymphocytaire, la TDM cérébrale ne montre en général aucune anomalie parenchymateuse. L'IRM cérébrale particulièrement les séquences FLAIR et éventuellement l'IRM médullaire sont incontournables dans la démarche diagnostique afin d'instaurer le traitement adéquat, permettant de mettre en évidence des lésions multifocales de la substance blanche sus et sous tentoriel, la prise de contraste est variable, et la séquence de diffusion contribue peu au diagnostic. La SEP reste le principal différentiel de l'ADEM, La distinction entre les 2 entités est souvent difficile et, parfois, seule l'épreuve du temps la permet. Le traitement de l'ADEM est basé sur les corticoïdes forts doses, éventuellement associés aux immunoglobulines polyvalentes ou aux Échanges plasmatiques. Le pronostic est généralement favorable sous traitement, cet élément souligne la nécessité de ne pas méconnaître ce diagnostic devant un tableau d'encéphalopathie inexpliquée.

### Etat des connaissances actuelles sur le sujet

- L'encéphalomyélite aiguë disséminée est une maladie inflammatoire démyélinisante multifocale du système nerveux central;
- Mécanisme est auto-immun suite à une vaccination ou à un épisode infectieux le plus souvent viral;
- L'IRM cérébrale montre des lésions diffuses ou multifocales de la substance blanche du SNC.

### Contribution de notre étude à la connaissance

- Le signe clinique le plus fréquent de l'ADEM est le déficit moteur chez l'adulte jeune et l'enfant;
- L'IRM cérébrale montre des lésions multifocales de la substance blanche en hyper signal T2 FLAIR sus sous tensoriel et de la moelle épinière;
- L'évolution clinique et radiologique est généralement favorable.

## Conflits des intérêts

Les auteurs ne déclarent aucun conflit d’intérêts.
